# Absence of causal relationship between Parkinson’s disease and subsequent prostate cancer: evidence from meta-analysis and Mendelian randomization studies

**DOI:** 10.3389/fonc.2023.1323812

**Published:** 2024-01-04

**Authors:** Li Wang, Jing-ya Deng, Xi-yue Fan, Dan Yang, Ping-yu Zhu, Xiao-ming Wang

**Affiliations:** ^1^ Department of Urology, Affiliated Hospital of North Sichuan Medical College, Nanchong, China; ^2^ Department of Neurology, Affiliated Hospital of North Sichuan Medical College, Nanchong, China; ^3^ Department of Neurology, Institute of Neurological Diseases of North Sichuan Medical College, Sichuan, Nanchong, China

**Keywords:** Parkinson’s disease, prostate cancer, Mendelian randomization, genetic variants, meta-analysis

## Abstract

**Background:**

Numerous observational studies have investigated the risk of prostate cancer (PCa) in patients diagnosed with Parkinson’s Disease (PD). However, the existence of a definitive association remains uncertain.

**Methods:**

Systematic searches were performed on PubMed, Web of Science, Scopus, and Google Scholar for studies published up to October 1, 2023. For Mendelian randomized (MR) causal inference, we employed pooled data from the IPDGC and PRACTICAL Consortium. The inverse variance weighted (IVW) method served as the principal technique for estimating odds ratios (ORs) and 95% confidence intervals (CIs) for the associations under investigation.

**Results:**

Cumulative analysis of nine studies revealed no significant association between patients diagnosed with PD and the subsequent incidence of PCa ([relative ratio] RR = 0.89, 95%CI = 0.73 to 1.08, P = 0.237). However, subgroup analyses indicated a reduced occurrence of PCa in Caucasian patients with PD (RR = 0.81, 95%CI = 0.69 to 0.95, P = 0.011). MR analyses failed to establish a significant link between increased genetic susceptibility to PD and the risk of PCa (IVW OR = 1.025, 95%CI = 0.997 to 1.054, P = 0.082). Sensitivity analyses further corroborated the robustness of these results.

**Conclusion:**

Both observational meta-analysis and MR analysis based on genetic variation do not support an association between PD patients and the subsequent risk of PCa. Further research is warranted to unravel the potential underlying mechanisms linking these two diseases.

**Systematic review registration:**

https://www.crd.york.ac.uk/PROSPERO/, identifier CRD42023473527.

## 1 Introduction

Parkinson’s disease (PD) is the second most common neurodegenerative disorder and increases with age (1). In individuals with PD, there is a loss of dopaminergic neurons in the substantia nigra pars compacta, leading to resting tremors, rigidity, motor dysfunction, and postural instability (2). Numerous cellular pathways, including mitochondrial dysfunction, excitotoxicity, compromised autophagic processes, oxidative stress, the accumulation of misfolded proteins, and genetic mutations, have been postulated as interlinked contributors to the neurodegenerative processes observed in PD (3).

Epidemiological evidence reveals a noteworthy correlation between PD and cancer (4–6). One hallmark of tumors is unbridled cell proliferation and a deficiency in apoptosis, whereas individuals with PD exhibit an augmented inclination toward cellular apoptosis (7). Certain studies postulate shared genetic and biological pathways between PD and cancer. Conversely, males demonstrate greater susceptibility to PD, implying a hormonal regulatory influence on PD (8). On the other hand, prostate cancer (PCa), as the second most common malignancy worldwide, is regulated by sex hormones and ranks as the sixth leading cause of cancer-related deaths in males (9). Previous studies on the incidence of PCa in patients with PD have yielded contentious outcomes (4, 10, 11), and observational studies cannot infer a causal relationship between PD and prostate cancer, as this might be influenced by reverse causation or confounding factors.

Mendelian randomization (MR) emerges as a method of instrumental variable (IV) analysis that harnesses single nucleotide polymorphisms (SNPs) derived from genome-wide association studies (GWAS) as tools to deduce causal associations between two traits (12). MR approximates the inherent attributes of a RCT and exhibits a reduced susceptibility to the impact of covariates. Moreover, its operational simplicity and cost-effectiveness enhance its appeal (13). Consequently, we conducted an updated meta-analysis and integrated MR studies to investigate the causal relationship between PD and PCa.

## 2 Methods

### 2.1 Meta-analysis

This study adheres to the Preferred Reporting Items for Systematic Review and Meta-Analysis (PRISMA) guidelines (**Supplementary Table 1**) and has been registered with PROSPERO (CRD42023473527) (14).

### 2.2 Search strategy

We conducted a comprehensive search of the published literature for associations between PD and prostate cancer in MEDLINE via the Cochrane Library, PubMed, Web of Science, Scopus and Google Scholar databases, up to October 1, 2023. The following strings were constructed using a combination of medical subject terms and keywords: [(Parkinson OR Parkinson disease OR PD) AND (prostate cancer OR prostate carcinomas OR prostate neoplasm)].

### 2.3 Eligibility criteria

Inclusion criteria were defined as follows: (1) Population-based study of patients with diagnostic criteria for PD. (2) Cohort or case-control studies of PD diagnosis prior to PCa; (3) studies that reported either an odds ratio (OR), relative risk (RR), hazard ratio (HR), or standardized incidence ratio (SIR) along with the corresponding confidence interval (CI); (3) original research published in English. The exclusion criteria comprised: (1) studies lacking relevant exposures (PD) and outcomes (Pca); (2) studies without meta-analysis data; (3) reviews, letters, case reports or conference reports. If study populations overlap, select the newest or most informative published studies.

### 2.4 Data acquisition and quality evaluation

Two investigators (JY, WL) employed EndNote X9 to identify and remove duplicate records. They subsequently reviewed both the titles and full texts of the remaining records for further screening. Relevant data were extracted and recorded in an Excel spreadsheet, including the following information: first author, year of publication, geographical region, duration of follow-up, method of PD diagnosis, number of cases and controls, adjusted covariates, risk values for outcome estimates. Two reviewers (XY and YD) evaluated the risk of bias using the Cochrane Collaboration Risk of Bias in Non-Randomized Studies of Interventions (ROBINS-I) tool (15). Moreover, we assessed study quality using the Newcastle-Ottawa Scale for cohort and case-control studies, with scores ranging from 0 to 9 (16). The included studies were categorized into two groups based on their mean quality score: a low-quality group (<7) and a high-quality group (≥7). In addition, the level of evidence (LOE) was graded according to the criteria of the Oxford Centre for Evidence-Based Medicine (17). In cases of disagreements, these were resolved through negotiation.

### 2.5 Statistical analysis

Given the low absolute incidence of prostate cancer, the four types of measurements were estimated to have similar RR values. In conjunction with previously published meta-analyses, we present the results using RR (18, 19). Due to the unavoidable high degree of heterogeneity between publications (P < 0.05, I^2^ > 50%), pooled effect sizes were calculated using random effects models. Otherwise, a fixed-effects model was used (P > 0.5, I^2^ < 50%). Egger’s test and funnel plots were utilized to evaluate publication bias. Sensitivity analyses assess the reliability of results by removing each study in turn. Furthermore, we performed subgroup analyses considering time to cancer diagnosis, study type, study quality, population, and year of publication. Meta-analyses were conducted using Stata 16.0 and considered statistically significant at p < 0.05.

### 2.6 Mendelian randomization

**Figure 1:**
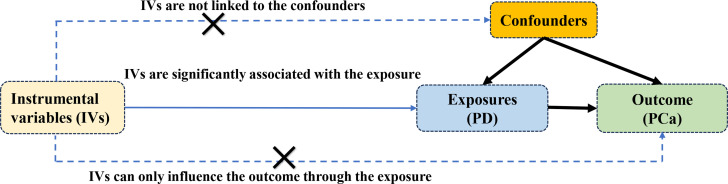
The three main assumptions of Mendelian randomization.

The study rigorously adhered to the guidelines outlined in the Strengthening the Reporting of Observational Studies in Epidemiology Mendelian Randomization (STROBE-MR) framework (20). MR relies on three essential assumptions: IVs demonstrate strong correlation with PD, remain unaffected by confounding variables, and impact Pca solely through the exposure under investigation. The basic assumptions and MR design flow are depicted in **Figure 1**. Since publicly available pooled data were utilized, ethical approval was not necessary for this study.

### 2.7 Data source and SNP selection

Summary data for PD were obtained from the comprehensive GWAS meta-analysis conducted by the International Parkinson’s Disease Genomics Consortium (IPDGC), encompassing 33,674 cases and 449,056 controls of European descent (21). GWAS data for Pca from Prostate Cancer Association Group to Investigate Cancer Associated Alterations in the Genome (PRACTICAL) Consortium (79,148 cases and 61,106 control cases) (22). To ensure the stability of the causal relationship between exposure and outcome, IVs were selected based on the following principles: (1) We established genome-wide significance thresholds for PD at p < 5×10^-8^. (2) Cluster analysis was conducted to address linkage disequilibrium (LD) among the selected IVs (r^2^ < 0.001, kb = 10,000). (3) Only SNPs with a minor allele frequency (MAF) exceeding 0.01 were considered. (4) To mitigate bias from weak IVs. the strength of the IVs was quantified using the F value (β^2^/SE), with those having F < 10 being excluded (23). Here, β represents the effect size of exposure and SE represents the standard error of the effect size. we also used Phenoscanner to examine potential confounders (such as body mass index, smoking, alcohol consumption and vitamin D supplementation) (24) (**Table 1**).

**Table 1:** Characteristics of the included GWAS summary studies in Mendelian randomization.

Trait	First author	Consortium	Sex/population	Sample size	Number of (cases/controls)	Year	GWAS ID
Exposure							
Parkinson’s disease	Nalls MA	IPDGC	Male and female/European	482,730	33,674/449,056	2019	ieu-b-7
Outcome							
Prostate cancer	Schumacher	PRACTICAL	Male/European	140,254	79,148/61,106	2018	ebi-a-GCST006085
Confounders							
Obesity	Berndt SI	GIANT	Male and female/European	98,697	32,858/65,839	2013	ieu-a-90
Smoking status: Current	Neale	Neale Lab	Male and female/European	336,024	33,928/302,096	2017	ukb-a-225
Ever smoked	Ben Elsworth	MRC-IEU	Male and female/European	461,066	280,508/180,558	2018	ukb-b-20261
Former alcohol drinker	Ben Elsworth	MRC-IEU	Male and female/European	31,506	16,191/15,315	2018	ukb-b-12654
Triglycerides	Willer CJ	GLGC	Males and females/Mixed	177,861	NA	2013	ieu-a-302
Vitamin D supplements	Ben Elsworth	MRC-IEU	Male and female/European	460,351	17,879/442,472	2018	ukb-b-12648

GWASs, genome-wide association studies; IPGDC, International Parkinson’s Disease Genomics Consortium; PRACTICAL, Prostate Cancer Association Group to Investigate Cancer Associated Alterations in the Genome; GIANT, genetic investigation of anthropometric traits consortium; MRC-IEU, MRC Integrative Epidemiology Unit; GLGC, Global Lipids Genetics Consortium; NA, not available.

### 2.8 Statistical analysis

The primary analysis employed the robust inverse-variance weighted (IVW) method (25). This method has the strongest statistical efficacy, but it must be satisfied that all genetic variation is a valid instrumental variable, and therefore we employed the weighted median, MR-Egger regression, maximum likelihood and simple weighted mode methods as validation approaches (26, 27). Sensitivity analysis assumes a vital role in the assessment of heterogeneity and potential biases within MR studies. Firstly, heterogeneity was evaluated through the application of Cochran’s Q test, which involved calculating the weighted sum of squared differences between specific variability estimates and the overall IVW estimate (28). To address potential outliers, the MR Pleiotropy RESidual Sum and Outlier (MR-PRESSO) method was employed during data analysis (29). Furthermore, MR-Egger regression was utilized, and intercepts were assessed to identify potential horizontal pleiotropy (p < 0.05 was judged significant). in addition, we performed a leave-one-out analysis to test the stability of the results. We evaluated heterogeneity among variant-specific causal estimates and pinpointed outliers through scatter and funnel plots. Finally, we identified potential bidirectional links between SNPs related to the PD and PCa using the MR Steiger Filtering Test (30). In addition, we performed multivariate MR (MVMR) analyses to observe the effect of confounding factors on PCa.

Statistical analyses were executed using R version 4.2.2 with the “TwoSampleMR” and “MRPRESSO” packages. Odds ratios (ORs) with 95% confidence intervals (CIs) were used to quantify the MR analysis, and statistical significance was defined as P < 0.05.

## 3 Result

### 3.1 Meta−analysis results

#### 3.1.1 Study characteristics and quality evaluation

**Figure 2:**
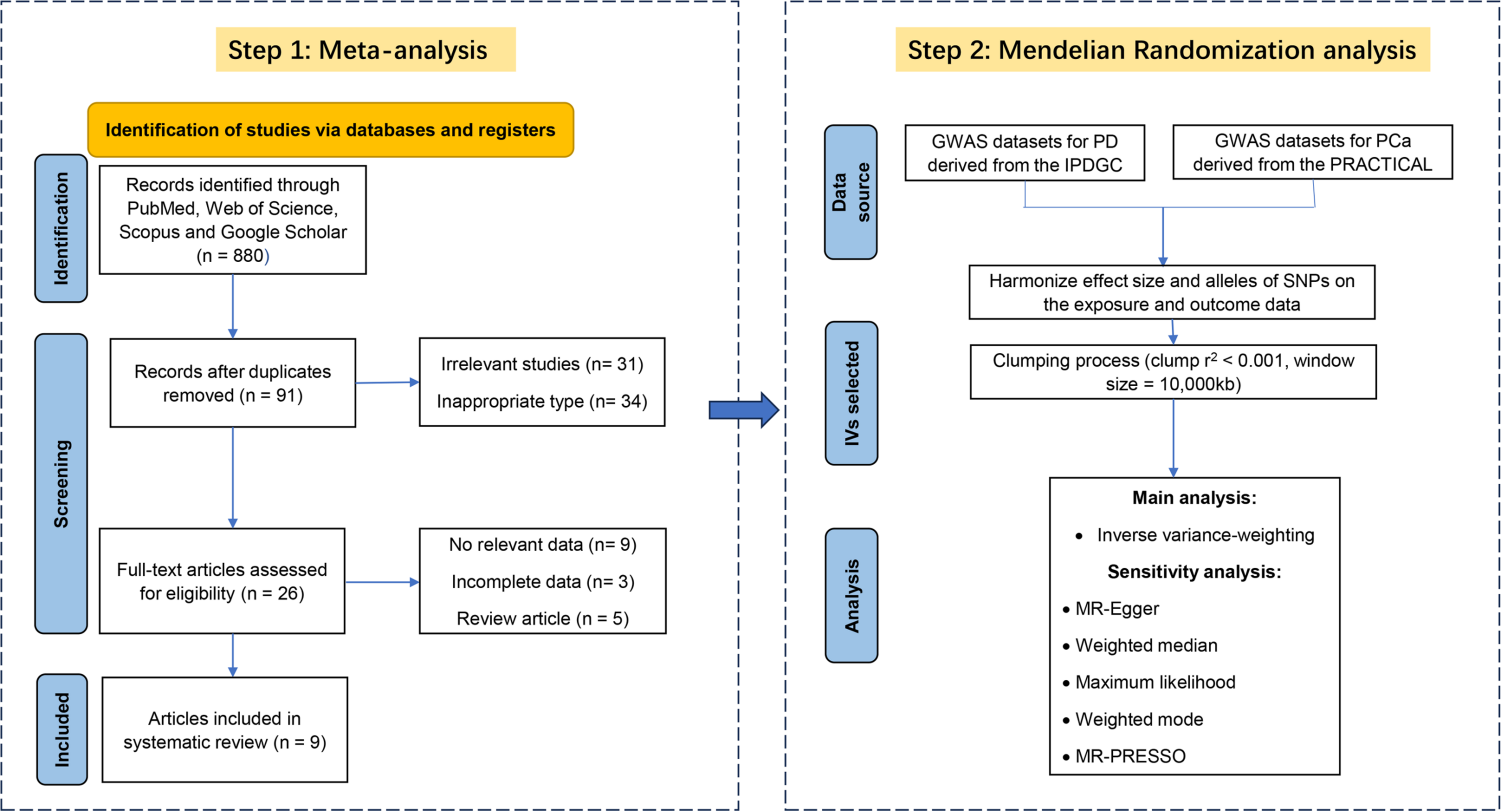
Flow chart for meta-analysis and Mendelian randomization analysis.

After a rigorous examination of online databases, 9 articles (5, 10, 31–37)(8 cohort and 1 case-control) from 2007 to 2019 were included in the final analysis. **Figure 2** illustrates the selection process, and **Table 2** provides detailed information on the included literature. 6 studies received high-quality ratings. however, all studies were at low to moderate risk of bias (**Supplementary Table 2**).

**Table 2:** Baseline characteristics of the included studies.

Author (year)	Design	Country	Mean or median follow-up (years)	Disease ascertainment	Sample size	Adjustment for covariates	Outcomes	NOS	LOE
Fois (31) (2010)	Cohort	UK	3.2	Coded	4,355 cases	Age, Sex, Time period in single calendar years and district of residence	RR	7	2b
Lo (32) (2010)	Cohort	UK	4.3	Medical record and clinical	692 cases; 761 controls	Age, sex, cigarette smoking, alcohol consumption, BMI	OR	7	2b
Wirdefeldt (33) (2014)	Cohort	Sweden	NA	Coded	11,786 cases; 58,930 controls	Age, sex, urbanization	HR	6	2b
Becker (34) (2010)	Case-control	UK	NA	Medical records	466 cases; 1864 controls	Age, sex, calendar time, BMI, smoking status	OR	9	2b
Driver (35) (2007)	Cohort	USA	5.2	Self- report PD diagnosis	572 cases; 478 controls	Smoking history, alcohol use, physical activity, BMI	RR	8	2b
Rugbjerg (36) (2012)	Cohort	Denmark	5.7	Coded	20343 cases	NA	SIR	6	2b
Ong (10) (2014)	Cohort	UK	12	Coded	219,194 cases; 9,015,614 controls	Age, sex, calendar year of first recorded admission, region of residence, quintile of patients’ Index of Deprivation score	RR	8	2b
Lin (5) (2015)	Cohort	Taiwan	7	Coded	62,023 cases; 124,046 controls	Age, sex	HR	6	2b
Park (37) (2019)	Cohort	Korea	6	Coded	52,009 cases; 260,045 controls	Age, sex, hypertension, diabetes mellitus, hyperlipidemia, income	HR	8	2b

BMI, body mass index; HR, hazard ratio; OR, odds ratio; PD, Parkinson disease; RR, relative risk; SIR, standardized incidence ratio; NA, not applicable.

#### 3.1.2 PCa risk in PD

**Figure 3:**
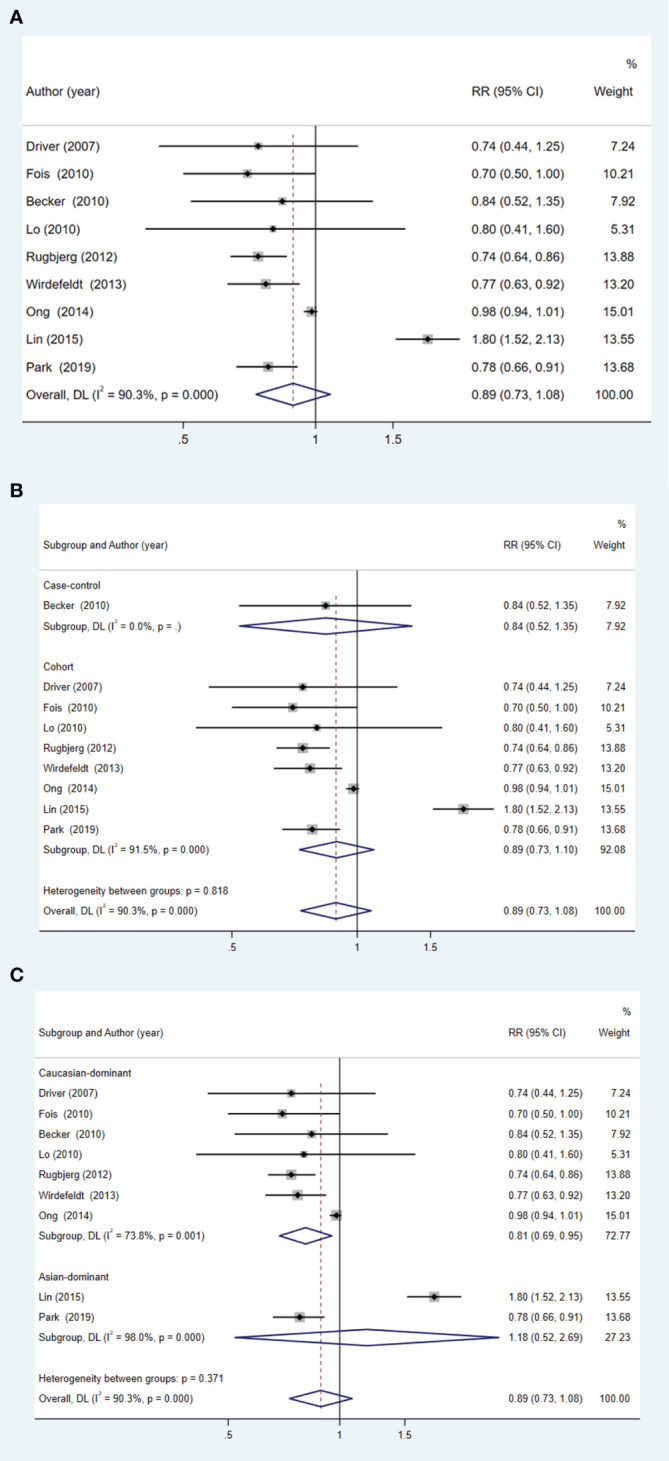
Forest plot of PCa risk in patients with PD and subgroup analysis. **(A)** overall effect; **(B)** subgroup analysis of study type; **(C)** subgroup analysis of different ethnicities.

Pooled analyses overall showed no significant association between patients with PD and the subsequent risk of PCa (RR = 0.89; 95% CI: 0.73 to 1.08; p = 0.237) (**Figure 3A**). This result held true across different types of studies (**Figure 3B**). Interestingly, within the Caucasian population, patients with PD were found to have a lower risk of PCa (RR = 0.81; 95% CI: 0.69 to 0.95; p = 0.011) (**Figure 3C**).

#### 3.1.3 Sensitivity analysis

Summarized effects remain stable through the successive exclusion of each study (**Supplementary Figure 1**). Furthermore, evidence of significant bias was not found in funnel plots or through Egger’s (p = 0.963) and Begg’s test (p = 0.297) (**Supplementary Figure 2**).

### 3.2 Mendelian randomization results

The *a priori* calculation of statistical power was conducted meticulously (38). By setting α at 5%, we attained a substantial statistical power exceeding 80% in scenarios where the expected OR concerning PCa were either at or below 1.04 within the context of PD.

#### 3.2.1 Effect of PD on PCa

**Figure 4:**
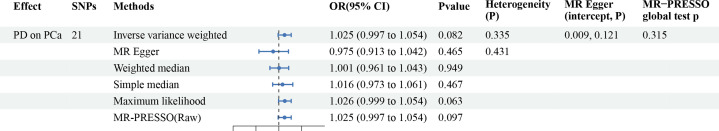
MR analysis results from PD to PCa risk.

The associations between the 21 designated SNPs and PCa are meticulously delineated in **Supplementary Table 3**. The range of variance expounded upon by these SNPs in relation to the exposure variables extended from 0.004 to 0.02. Furthermore, the IVs demonstrated robust statistical significance (F > 10). After a rigorous Steiger filtering process, no signs of reverse causality were found. There is no apparent association between genetic predisposition to PD and the occurrence of PCa (OR = 1.025; 95% CI: 0.997 to 1.054; P = 0.082), which is consistent with the overall effect results of the meta-analysis. No heterogeneity was observed in the sensitivity analysis, and there was no horizontal pleiotropy detected in the MR-Egger analysis. Additionally, the MR-PRESSO test did not identify any outliers (Global test p = 0.315) (**Figure 4**; **Supplementary Figure 3**). Results between genetic susceptibility to PD and PCa remained robust in MVMR adjusted for relevant confounders (**Table 3**).

**Table 3:** Complete MVMR results of PD in relevant prostate cancer risk factors.

Adjustments	Methods	Parkinson’s Disease					
		SNPs	Causal effect		Heterogeneity	Pleiotropy	
			OR (95% CI)	*P*	P*	Intercept	*P*
Obesity	IVW	22	1.05 (0.99-1.13)	0.11	0.066	0.005	0.199
	Median		1.01 (0.93-1.09)	0.83			
	Egger		1.05 (0.99-1.12)	0.12			
Smoking status: Current	IVW	35	1.00 (0.96-1.04)	0.87	< 0.001	0.003	0.522
	Median		1.01 (0.97-1.05)	0.75			
	Egger		0.98 (0.91-1.06)	0.65			
Ever smoked	IVW	90	1.01 (0.98-1.04)	0.60	< 0.001	0.002	0.290
	Median		1.02 (0.98-1.07)	0.27			
	Egger		0.99 (0.95-1.04)	0.81			
Former alcohol drinker	IVW	19	1.01 (0.96-1.06)	0.69	< 0.001	0.012	0.358
	Median		1.01 (0.96-1.05)	0.81			
	Egger		0.93 (0.78-1.11)	0.45			
Triglycerides	IVW	53	0.99 (0.90-1.10)	0.92	< 0.001	0.003	0.365
	Median		0.99 (0.92-1.08)	0.95			
	Egger		0.99 (0.89-1.10)	0.79			
Vitamin D supplements	IVW	19	1.01 (0.96-1.06)	0.70	< 0.001	0.010	0.445
	Median		1.04 (0.99-1.08)	0.10			
	Egger		0.95 (0.80-1.13)	0.54			

^*^Heterogeneity P < 0.05 indicated potential heterogeneity existing in the IVW model, and the median method was suggested for causal inference in this situation. MVMR, multivariable Mendelian randomization; SNP, single nucleotide polymorphisms; IVW, inverse variance weighted; OR, odds ratio; CI, confidence interval; PD Parkinson’s Disease.

## 4 Discussion

This study has undertaken a comprehensive assessment of the risk of PCa in patients diagnosed with PD. The results of cumulative analysis and MR analysis have confirmed the lack of significant correlation between PD and PCa under genetic prediction. The co-occurrence of two distinct diseases within the same individual may stem from shared environmental or genetic factors. Previous studies have yielded conflicting evidence regarding the relationship between PD and cancer (5, 37), and several potential explanatory mechanisms have been proposed.

PD, a neurodegenerative disorder, is characterized by the demise of dopaminergic neurons, distinguishing it from PCa, which is typified by unrestricted cellular proliferation and a lack of apoptosis. Interestingly, cells in PD patients exhibit a greater propensity to undergo apoptosis, which may serve as a defensive mechanism against cancer progression.

Smoking is recognized as a significant risk factor for various types of tumors while seemingly reducing the risk of developing PD (39). Nicotine has been observed to stimulate the release of dopamine and demonstrate neuronal protection in various experimental models (40). Although PCa is not typically associated with smoking, earlier investigations have reported a decreased risk of PCa among individuals with PD (4, 41). It’s worth noting that patients diagnosed with PD typically have higher mortality rates than the general population. Furthermore, those who do survive are less likely to die from subsequent cancers (42).

One of the therapeutic strategies for individuals with PD involves increasing dopamine levels within the central nervous system, thereby stimulating the sympathetic nerves. Concurrently, anticholinergic drugs might act on parasympathetic nerves to alleviate symptoms (43). The stroma of the prostate is heavily innervated by branches of the autonomic nervous system, which play a significant role in the growth and sustenance of the prostate gland (44). A study by Magnon et al. (45) discovered that sympathetic neurons foster tumor genesis at an early stage, while parasympathetic fibers drive the dissemination of cancer. Consequently, medications targeting branches of the autonomic nervous system could potentially offer therapeutic advantages.

Levodopa and other dopaminergic drugs may be administered following a diagnosis of PD. Current studies indicate that L-Dopa decarboxylase (DDC) is an androgen receptor co-activator, its expression increases with the progression of the disease, and its co-expresses with receptors in prostate cancer cells. The related drugs enhance anti-tumor activity by inhibiting the DDC pathway (46). Interestingly, our findings indicate that Caucasian populations exhibit a lower prevalence of PCa following the onset of PD. Lin et al. (5)discovered that Taiwanese men diagnosed with PD had an elevated risk of PCa, a phenomenon attributed to a confluence of distinctive genetic backgrounds, habits, and/or environmental exposures. However, in MR analyses conducted on European populations, no significant causal association was observed between PD and the risk of subsequent PCa occurrence. This appears to suggest that the results of meta-analyses may have been influenced by bias and confounding factors.

### 4.1 Strength and limitation

Our study possesses several strengths. Firstly, we adhered strictly to PRISMA guidelines in our literature screening and conducted subgroup analyses and bias assessments. Secondly, our MR study adhered to the three key hypotheses and utilized a two-sample approach to explore the causal relationship between PD and PCa. Sensitivity analyses confirmed the reliability of our results, while MVMR analyses helped to eliminate confounding bias. Despite these strengths, our study is not without limitations. For one, the MR analysis validated results solely for the European population, which might have resulted in a more homogeneous association. Furthermore, we did not perform a gender-stratified analysis, which may have introduced some bias. Moreover, the results of the meta-analysis were inevitably highly heterogeneous. Finally, the insufficient sample size may lead to instability in subgroup effects, and future studies with larger sample sizes are needed to enhance the reliability of the results.

## 5 Conclusion

This comprehensive MR and meta-analysis did not demonstrate an association between PD and PCa risk. The potential biological pathways contributing to the co-morbidity between these two diseases certainly warrant further exploration.

## Data availability statement

The original contributions presented in the study are included in the article/**Supplementary Material**. Further inquiries can be directed to the corresponding authors.

## Author contributions

LW: Writing – original draft. JD: Data curation, Writing – original draft. XF: Methodology, Writing – original draft. DY: Formal Analysis, Writing – original draft. PZ: Methodology, Supervision, Writing – review & editing. XW: Funding acquisition, Supervision, Validation, Writing – review & editing.
